# Novel insulin sensitizer MSDC-0602K improves insulinemia and fatty liver disease in mice, alone and in combination with liraglutide

**DOI:** 10.1016/j.jbc.2021.100807

**Published:** 2021-05-20

**Authors:** Dakota R. Kamm, Kelly D. Pyles, Martin C. Sharpe, Laura N. Healy, Jerry R. Colca, Kyle S. McCommis

**Affiliations:** 1 Biochemistry & Molecular Biology, Saint Louis University School of Medicine, St Louis, Missouri, USA; 2 LNH Tox Path Consulting LLC, Newbury Park, California, USA; 3 Cirius Therapeutics, Kalamazoo, Michigan, USA; 4 Cirius Therapeutics, San Diego, California, USA

**Keywords:** beta cell, diabetes, hyperinsulinemia, insulin resistance, insulin secretion, metabolism, NAFLD, NASH, ALT, alanine transaminase, AST, aspartate transaminase, DMEM, Dulbecco's modified Eagle's medium, DMSO, dimethyl sulfoxide, GLP-1RA, glucagon-like peptide-1 receptor agonist, GSIS, glucose-stimulated insulin secretion, Lira, liraglutide, MPC, mitochondrial pyruvate carrier, NAFLD, nonalcoholic fatty liver disease, NASH, nonalcoholic steatohepatitis, NEFA, nonesterified fatty acid, PPARγ, peroxisome proliferator-activated receptor γ, RIP, rat insulin promoter, TAG, triacylglyceride, TBST, Tris-buffered saline with Tween-20, TZD, thiazolidinedione, Veh, vehicle solution

## Abstract

Insulin sensitizers and incretin mimetics are antidiabetic agents with vastly different mechanisms of action. Thiazolidinedione (TZD) insulin sensitizers are associated with weight gain, whereas glucagon-like peptide-1 receptor agonists can induce weight loss. We hypothesized that combination of a TZD insulin sensitizer and the glucagon-like peptide-1 receptor agonist liraglutide would more significantly improve mouse models of diabetes and nonalcoholic steatohepatitis (NASH). Diabetic *db/db* and MS-NASH mice were treated with the TZD MSDC-0602K by oral gavage, liraglutide (Lira) by s.c. injection, or combination 0602K+Lira. Lira slightly reduced body weight and modestly improved glycemia in *db/db* mice. Comparatively, 0602K-treated and 0602K+Lira-treated mice exhibited slight weight gain but completely corrected glycemia and improved glucose tolerance. 0602K reduced plasma insulin, whereas Lira further increased the hyperinsulinemia of *db/db* mice. Surprisingly, 0602K+Lira treatment reduced plasma insulin and C-peptide to the same extent as mice treated with 0602K alone. 0602K did not reduce glucose-stimulated insulin secretion *in vivo*, or in isolated islets, indicating the reduced insulinemia was likely compensatory to improved insulin sensitivity. In MS-NASH mice, both 0602K or Lira alone improved plasma alanine aminotransferase and aspartate aminotransferase, as well as liver histology, but more significant improvements were observed with 0602K+Lira treatment. 0602K or 0602K+Lira also increased pancreatic insulin content in both *db/db* and MS-NASH mice. In conclusion, MSDC-0602K corrected glycemia and reduced insulinemia when given alone, or in combination with Lira. However, 0602K+Lira combination more significantly improved glucose tolerance and liver histology, suggesting that this combination treatment may be an effective therapeutic strategy for diabetes and NASH.

Insulin resistance can result in elevated rates of insulin secretion and hyperinsulinemia. If unresolved, pancreatic beta cell failure can lead to loss of beta cell mass *via* cell death or dedifferentiation (1, 2). Insulin sensitizers are an attractive therapeutic strategy as they not only target the core defective insulin signaling pathways but can also reduce this stress on beta cells and preserve beta cell mass (3). However, clinical use of the main class of insulin sensitizers, the thiazolidinediones (TZDs), is limited owing to side effects such as weight gain, edema, bone loss, and bladder cancer risk. These side effects are thought to be due to agonism of the nuclear receptor peroxisome proliferator-activated receptor γ (PPARγ). Yet numerous studies have described acute, PPARγ-independent effects of TZDs (4, 5, 6, 7), which have led to the development of several TZDs with very low affinity for PPARγ, such as MSDC-0602K, MSDC-0160, and PXL065. The molecular target of these compounds is the mitochondrial pyruvate carrier (MPC) (8, 9, 10), which is also inhibited by the traditional PPARγ-activating TZDs (9). The clinical profile of PPARγ-sparing TZDs appears improved compared with traditional TZDs with respect to edema, bone loss, and degree of weight gain (11, 12, 13).

Another popular class of antidiabetic agents is the glucagon-like peptide-1 receptor agonists (GLP-1RA). These incretin-like peptides improve glycemia by increasing postprandial insulin secretion, suppressing glucagon secretion, and delaying gastric emptying. GLP-1RAs such as liraglutide can also induce weight loss in humans and animal models (14, 15).

Insulin resistance and diabetes are driving factors for the development and progression of nonalcoholic fatty liver disease (NAFLD) and nonalcoholic steatohepatitis (NASH). Although there are currently no approved therapies for treatment of NAFLD/NASH, both TZDs (13, 16, 17, 18, 19, 20, 21) and GLP-1RAs (22, 23, 24, 25) can improve aspects of liver pathology in humans and animal models. The purpose of this current study was to investigate whether combining the PPARγ-sparing TZD MSDC-0602K and the GLP-1RA liraglutide would better improve mouse models of diabetes and NASH.

## Results

### Effects of MSDC-0602K and liraglutide on body weight, tissue weights, and glycemia of *db/db* mice

**Figure 1:**
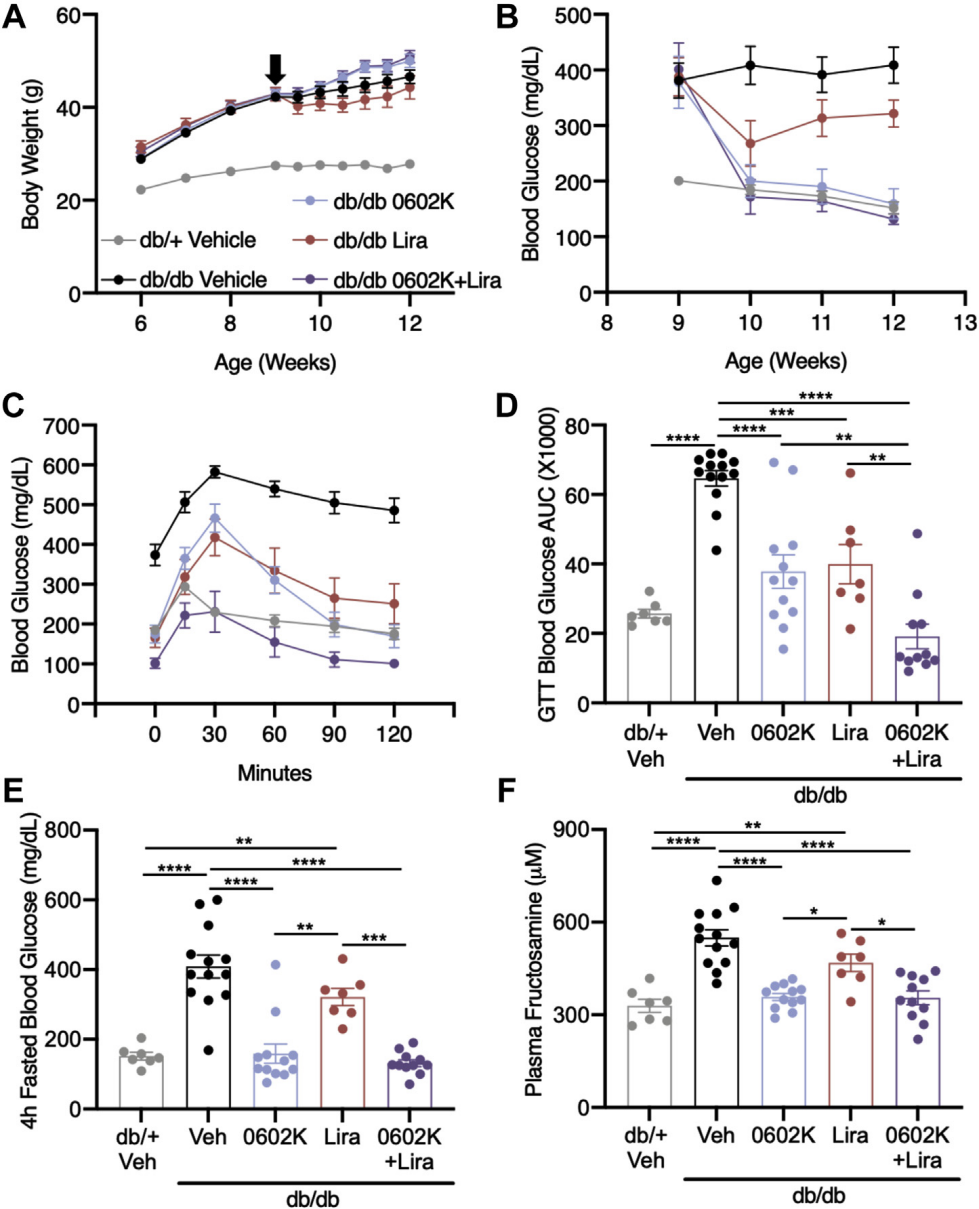
**MSDC-0602K alone and in combination with liraglutide improves glycemia and glucose tolerance in *db/db* mice.***A*, average body weights of each treatment group. *B*, average weekly blood glucose concentrations. *C* and *D*, blood glucose excursions and calculated area under the curve from an i.p. glucose tolerance test (GTT). *E*, 4-h fasted blood glucose concentrations measured at time of sacrifice. *F*, plasma fructosamine concentrations. N = 7 *db/+* Veh; 13 *db/db* Veh, 12 *db/db* 0602K, 7 *db/db* Lira, and 11 *db/db* 0602K+Lira. All data are mean ± SEM. Individual data points represent a single mouse. Ordinary one-way ANOVA with Tukey’s multiple comparison test: ∗*p* < 0.05, ∗∗*p* < 0.01, ∗∗∗*p* < 0.001, and ∗∗∗∗*p* < 0.0001.

Beginning at 9 weeks of age, *db/db* mice were treated with MSDC-0602K, liraglutide, combination, or both vehicle solutions. 0602K treatment caused slight weight gain and Lira treatment caused slight weight loss compared with vehicle-treated *db/db* mice (Fig. 1*A*). Mice treated with 0602K+Lira combination displayed slight weight gain similar to mice treated with 0602K alone (Fig. 1*A*). Blood glucose was monitored weekly and was completely corrected to *db/+* levels by 0602K or 0602K+Lira treatments, whereas Lira treatment more modestly improved glycemia (Fig. 1*B*). Both drugs individually improved glucose tolerance compared with vehicle-treated *db/db* mice, and the 0602K+Lira combination improved glucose tolerance beyond even lean *db/+* mouse levels (Fig. 1, *C* and *D*). At sacrifice, 4-h fasted blood glucose levels were completely corrected by 0602K or 0602K+Lira (Fig. 1*E*). Plasma fructosamine levels supported the weekly glucose measurements indicating that Lira modestly improved glycemia, whereas 0602K or 0602K+Lira completely corrected glycemia (Fig. 1*F*). These results indicate that, although 0602K alone is able to correct glycemia, the combination of 0602K+Lira better improves glucose tolerance in *db/db* mice.

**Figure 2:**
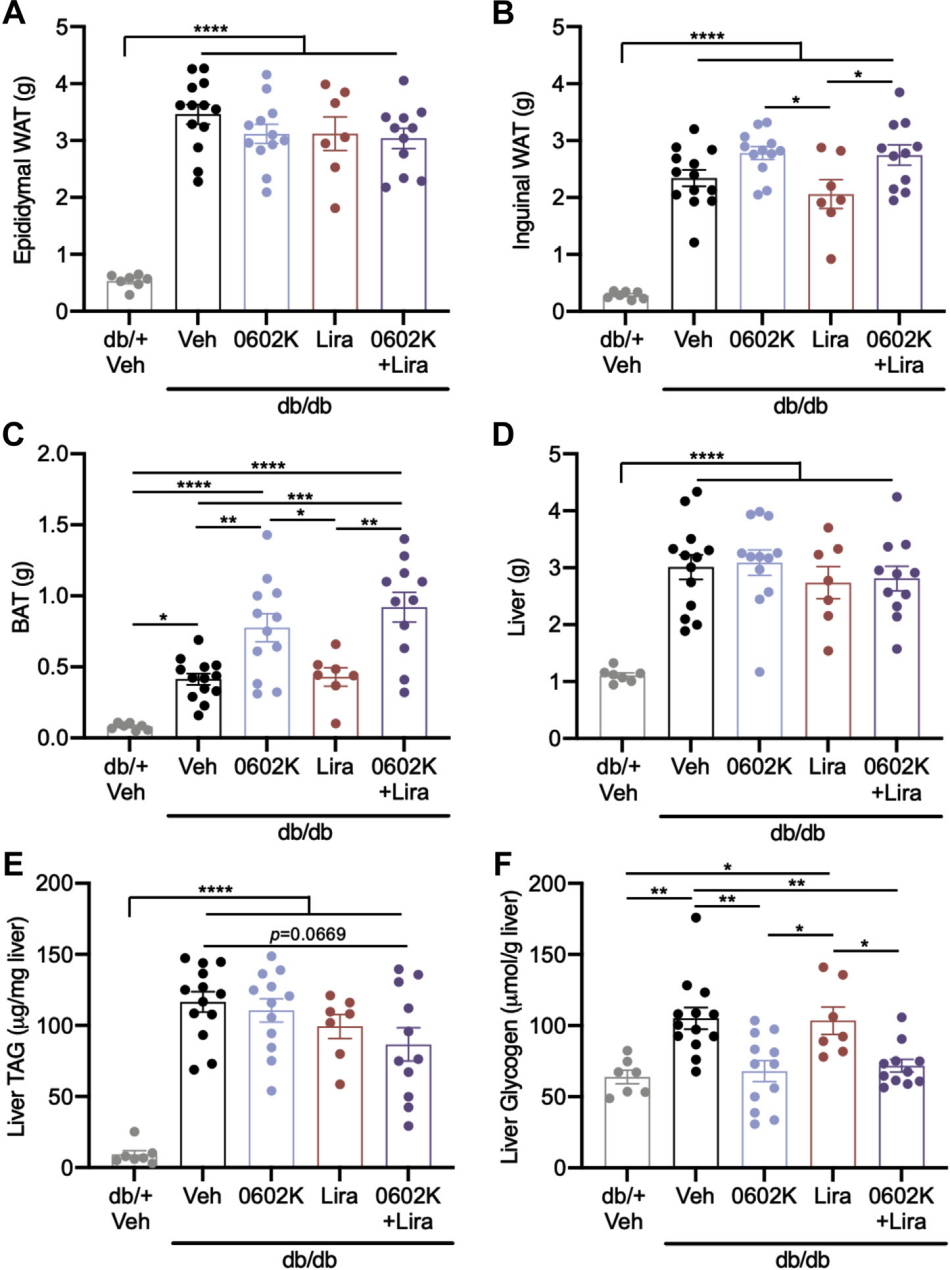
**Effects of MSDC-0602K or liraglutide on tissue weights, liver triglycerides, and glycogen.***A*–*D*, epididymal (visceral) white adipose tissue (WAT), inguinal (subcutaneous) WAT, intrascapular brown adipose tissue (BAT), and liver weights. *E* and *F*, hepatic triglyceride (TAG) and glycogen concentrations. N = 7 *db/+* Veh; 13 *db/db* Veh, 12 *db/db* 0602K, 7 *db/db* Lira, and 11 *db/db* 0602K+Lira. All data are mean ± SEM. Individual data points represent a single mouse. Ordinary one-way ANOVA with Tukey’s multiple comparison test: ∗*p* < 0.05, ∗∗*p* < 0.01, ∗∗∗*p* < 0.001, and ∗∗∗∗*p* < 0.0001.

Epididymal white adipose tissue weights were unaffected by drug treatment (Fig. 2*A*), but 0602K trended to increase inguinal subcutaneous white adipose tissue and significantly increased brown adipose tissue weight whether provided alone or in combination with Lira (Fig. 2, *B* and *C*). Increased liver weights in *db/db* mice were not altered by drug treatment (Fig. 2*D*), yet hepatic triacylglyceride (TAG) was nearly significantly reduced by 0602K+Lira (Fig. 2*E*). Hepatic glycogen levels were also increased in *db/db* mice and were completely normalized by either 0602K or 0602K+Lira (Fig. 2*F*). Thus, 0602K increased adiposity and corrected hepatic glycogen levels whether given alone or in combination with Lira.

### Effects of MSDC-0602K and liraglutide on insulinemia and plasma lipids

**Figure 3:**
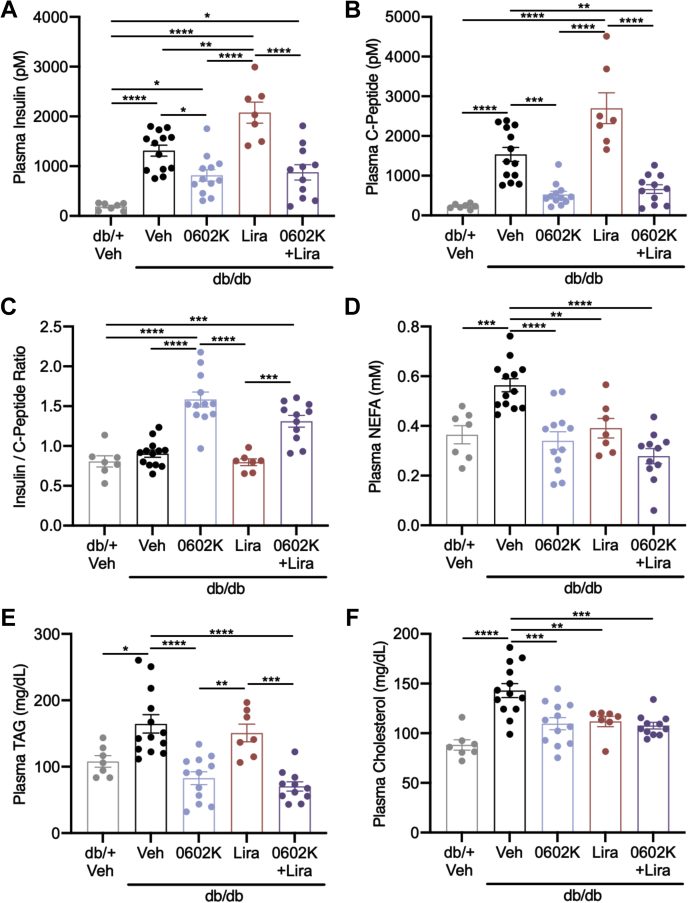
**MSDC-0602K reduces insulinemia and plasma lipids.***A*–*F*, plasma insulin, C-peptide, insulin/C-peptide ratio, nonesterified fatty acids (NEFA), triglycerides (TAG), and cholesterol concentrations. N = 7 *db/+* Veh; 13 *db/db* Veh, 12 *db/db* 0602K, 7 *db/db* Lira, and 11 *db/db* 0602K+Lira. All data are mean ± SEM. Individual data points represent a single mouse. Ordinary one-way ANOVA with Tukey’s multiple comparison test: ∗*p* < 0.05, ∗∗*p* < 0.01, ∗∗∗*p* < 0.001, and ∗∗∗∗*p* < 0.0001.

Vehicle-treated *db/db* mice were hyperinsulinemic, and 0602K decreased these plasma insulin concentrations (Fig. 3*A*). In contrast, Lira treatment further increased plasma insulin, yet mice treated with 0602K+Lira displayed reduced insulin similar to 0602K-treated animals (Fig. 3*A*). To resolve whether the changes in insulinemia were due to insulin secretion *versus* insulin clearance, we measured plasma C-peptide concentrations, which were strongly elevated in vehicle-treated *db/db* mice. Although Lira further increased C-peptide concentrations, 0602K or 0602K+Lira-treated mice displayed reduced C-peptide (Fig. 3*B*). An increased insulin/C-peptide ratio in 0602K-treated mice suggests that 0602K more strongly reduced insulin secretion *versus* increased clearance (Fig. 3*C*). Plasma nonesterified fatty acids (NEFAs), TAG, and cholesterol were all increased in vehicle-treated *db/db* mice, and reduced by 0602K, Lira, or 0602K+Lira treatments (Fig. 3, *D*–*F*). These results suggest that 0602K reduces hyperinsulinemia by reducing insulin secretion even in combination with liraglutide, yet all treatments were able to improve plasma lipids.

### MSDC-0602K improves insulin sensitivity and partially restores islet insulin content

**Figure 4:**
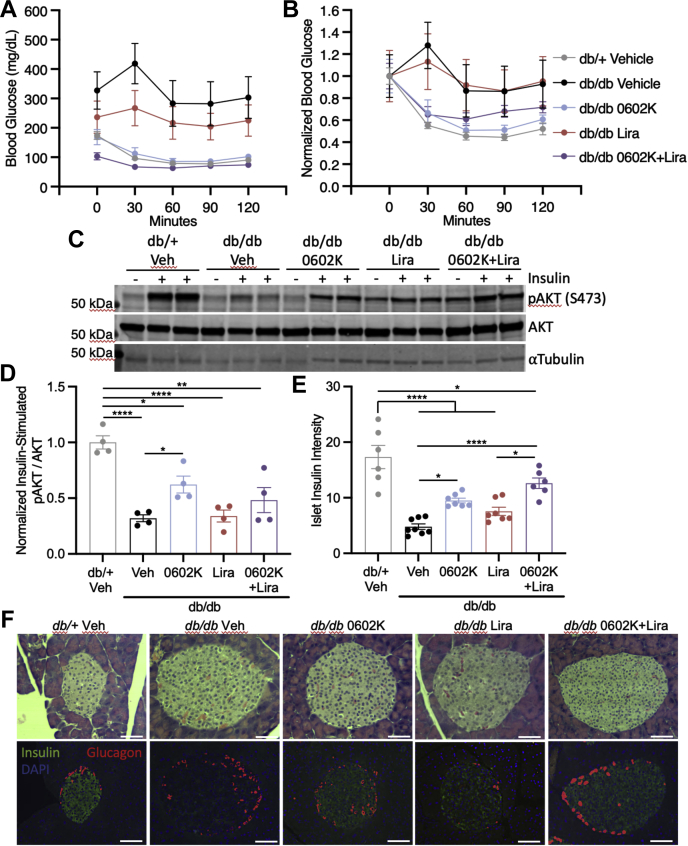
**MSDC-0602K improves insulin sensitivity and partially restores islet insulin content.***A* and *B*, blood glucose concentrations and glucose concentrations normalized to T = 0 values during an i.p. insulin tolerance test (n = 5). *C* and *D*, representative Western blot and quantified densitometry of liver lysates for insulin-stimulated AKT phosphorylation (n = 1 saline control and n = 4 insulin injection for all groups). *E* and *F*, representative H&E and insulin/glucagon immunofluorescence images of pancreatic islets (scale bars, 100 μm) with islet insulin intensity measured by the islet *green* fluorescence normalized to *green* fluorescence of surrounding exocrine pancreas tissue. Individual data points represent the average intensity of at least 12 islets per mouse. All data are mean ± SEM. Ordinary one-way ANOVA with Tukey’s multiple comparison test: ∗*p* < 0.05, ∗∗*p* < 0.01, and ∗∗∗∗*p* < 0.0001.

A subset of mice was subjected to an i.p. insulin tolerance test, which when normalized to starting glucose levels, clearly indicated that only *db/+* lean mice, or mice treated with 0602K, had a significant decrease in blood glucose from insulin (Fig. 4, *A* and *B*). Subsequently, mice were injected with insulin i.p. and sacrificed after 10 min to assess insulin signaling. Livers from *db/+* mice displayed a robust phosphorylation of AKT upon insulin injection, which was severely blunted in vehicle-treated *db/db* livers (Fig. 4, *C* and *D*). Although 0602K treatment partially restored the insulin-stimulated AKT phosphorylation in the liver, liraglutide treatment resulted in no improvement (Fig. 4, *C* and *D*). These improvements in insulin sensitivity led to improved pancreatic islet insulin content, which was very low in vehicle-treated *db/db* mice (Fig. 4, *E* and *F*). Altogether, these results suggest that 0602K treatment with or without liraglutide improves insulin sensitivity and partially restores islet insulin content.

### MSDC-0602K does not acutely inhibit insulin secretion

**Figure 5:**
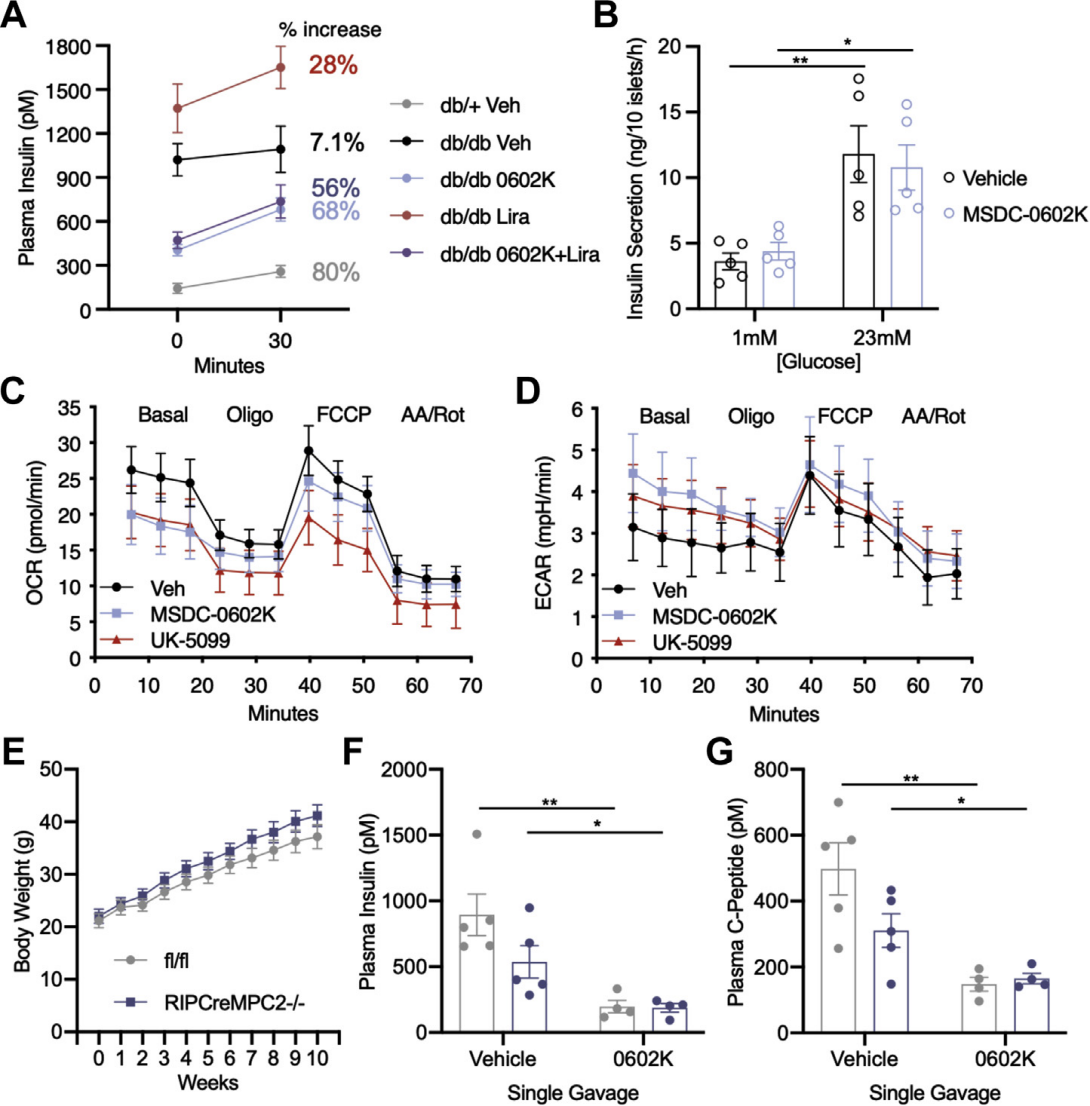
**MSDC-0602K does not acutely inhibit insulin secretion despite reducing β-cell pyruvate metabolism.***A*, plasma insulin before and 30 min after 1 g/kg i.p. glucose injection and calculated % increase of insulin concentration (n = 5). *B*, insulin secretion from isolated wildtype mouse islets at 1 *versus* 23 mM glucose comparing dimethyl sulfoxide vehicle with 10 μM MSDC-0602K treatment. Individual data points represent the average of an independent experiment, each containing 3 to 4 technical replicates. *C* and *D*, seahorse analysis of oxygen consumption rate (OCR) and extracellular acidification rate (ECAR) of beta-TC6 insulinoma cells pretreated for 1 h with dimethyl sulfoxide vehicle, 20 μM 0602K, or 5 μM UK-5099. *E*, average weekly body weights of fl/fl (wildtype) and beta cell–specific MPC2 knockout (RIPCreMPC2−/−) mice fed high-fat diet for 10 weeks. After 10 weeks of high-fat diet, mice were gavaged once with vehicle or 30 mg/kg MSDC-0602K and euthanized the following morning after ∼16 h. *F* and *G*, plasma insulin and C-peptide measured after a single dose of vehicle or 0602K 16 h prior. All data are mean ± SEM. Individual data points represent a single mouse. Ordinary one-way ANOVA with Tukey’s multiple comparison test: ∗*p* < 0.05 and ∗∗*p* < 0.01.

We next wanted to test whether 0602K was directly inhibiting insulin secretion. This is particularly intriguing as 0602K inhibits the MPC (9, 10), and we have previously shown that mice with beta cell–specific MPC deletion display defective glucose-stimulated insulin secretion (GSIS) (26). In a subset of mice, plasma insulin was measured before and 30 min after an i.p. injection of glucose. 0602K- or 0602K+Lira-treated mice displayed significantly lower starting insulinemia; however, these mice had ∼60% increases in insulin concentrations after glucose injection compared with 7% increase in vehicle-treated *db/db* mice (Fig. 5*A*). Although these results suggest that 0602K treatment does not directly inhibit insulin secretion, we also tested this in isolated wildtype mouse islets, which also showed no effect of 0602K treatment on GSIS (Fig. 5*B*). Of interest, 0602K does appear to inhibit the MPC in cultured Beta-TC6 insulinoma cells, as 0602K or the MPC inhibitor UK-5099 were both able to reduce glucose/pyruvate-stimulated oxygen consumption and increase glycolysis (Fig. 5, *C* and *D*). Therefore, despite inhibiting the beta cell MPC and mitochondrial pyruvate metabolism, 0602K does not acutely inhibit insulin secretion. Finally, a single gavaged dose of 0602K 16 h prior was able to reduce plasma insulin and C-peptide concentrations in diet-induced obese fl/fl (wildtype) and rat insulin promoter (RIP) Cre-driven beta cell MPC2 knockout mice (Fig. 5, *E*–*G*). Thus, insulin secretion was reduced by 0602K even when the molecular target of 0602K was not expressed in the pancreatic beta cells. Altogether, these results suggest that 0602K does not acutely inhibit insulin secretion but rather reduces insulin secretion chronically owing to improved peripheral insulin sensitivity.

### MSDC-0602K and liraglutide improve liver pathology in a mouse model of NASH

**Figure 6:**
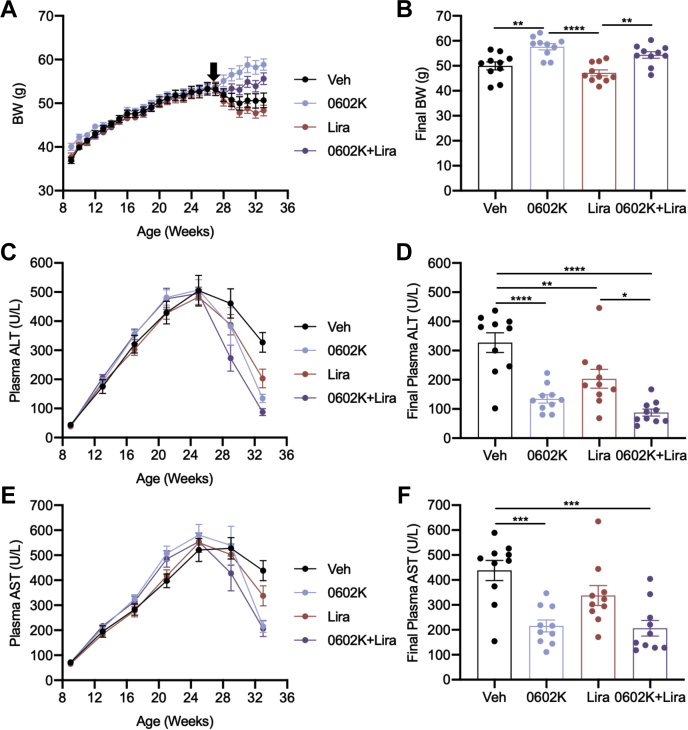
**MSDC-0602K and liraglutide improve plasma measures of liver injury.***A* and *B*, average body weight (BW) and final BW of MS-NASH mice treated with Vehicle, 0602K, liraglutide, or 0602K+Lira. *C*–*F*, average monthly plasma alanine aminotransferase (ALT) and aspartate aminotransferase (AST) and final ALT and AST. n = 10 for all groups. All data are mean ± SEM. Individual data points represent a single mouse. Ordinary one-way ANOVA with Tukey’s multiple comparison test: ∗*p* < 0.05, ∗∗*p* < 0.01, ∗∗∗*p* < 0.001, and ∗∗∗∗*p* < 0.0001.

**Figure 7:**
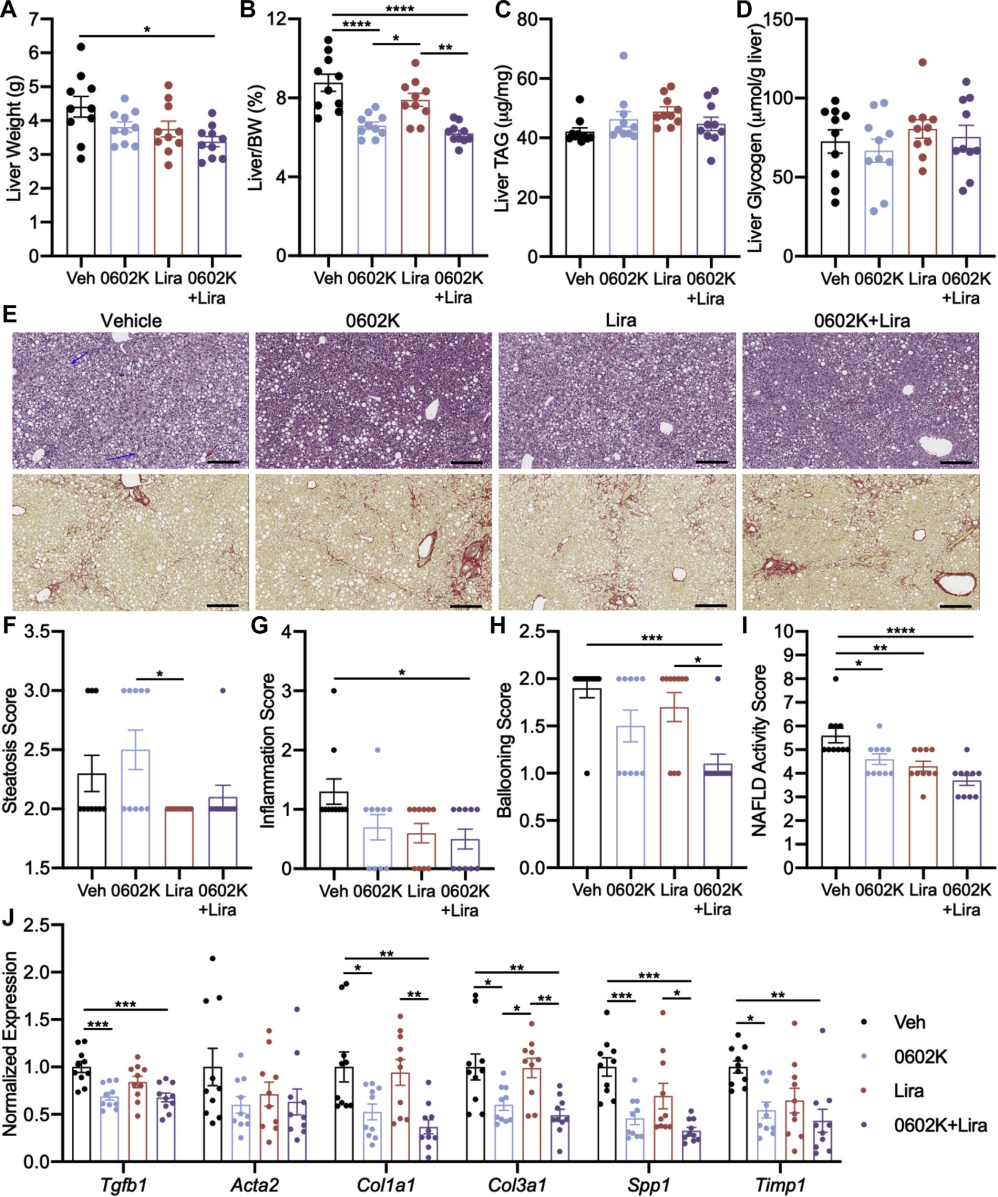
**MSDC-0602K+liraglutide treatment improves NASH pathology.***A* and *B*, liver weight and liver weight normalized to body weight (BW). *C* and *D*, liver triglyceride (TAG) and glycogen concentrations. *E*, representative liver H&E and picrosirius red images (scale bars, 200 μm). *F*–*I*, nonalcoholic fatty liver disease histology scores. *J*, hepatic gene expression for stellate cell activation, collagen, and extracellular matrix. n = 10 for all groups. All data are mean ± SEM. Individual data points represent a single mouse. Ordinary one-way ANOVA with Tukey’s multiple comparison test: ∗*p* < 0.05, ∗∗*p* < 0.01, ∗∗∗*p* < 0.001, and ∗∗∗∗*p* < 0.0001.

Insulin resistance is a strong driver of NAFLD, and 0602K improves aspects of NASH histology in both mice and humans (13, 16). To test if NASH was more significantly improved with dual 0602K and Lira therapy, MS-NASH mice were fed a Western diet and provided fructose in the drinking water to develop obesity and NASH. Vehicle, 0602K, Lira, or 0602K+Lira treatments were started after 18 weeks on diet, and similar to the *db/db* study, 0602K-treated mice tended to display increased body weight (Fig. 6, *A* and *B*). Plasma alanine aminotransferase (ALT) and aspartate aminotransferase (AST) were monitored monthly, and Lira caused modest reductions in these plasma markers of liver injury, whereas 0602K or 0602K+Lira induced more significant reductions (Fig. 6, *C*–*F*). Only 0602K+Lira reduced liver weights compared with vehicle-treated mice; however, as a percentage of body weight, 0602K- or 0602K+Lira-treated mice displayed reduced liver size (Fig. 7, *A* and *B*). Liver TAG and glycogen concentrations were unaffected by any treatment in these mice (Fig. 7, *C* and *D*). Scoring of liver histology agreed with the biochemical measurements that steatosis was not improved by any treatment (Fig. 7, *E* and *F*). However, hepatic inflammation and ballooning were significantly improved by combination 0602K+Lira treatment (Fig. 7, *G* and *H*). 0602K or Lira alone improved the NAFLD activity score, whereas the 0602K+Lira combination more significantly improved NASH liver histology (Fig. 7, *E* and *I*). Fine bridging fibrosis was identified in all mice, and although the fibrosis histology scores were not improved by any treatment (data not shown), 0602K or 0602K+Lira treatment reduced the expression of several collagen and extracellular matrix–related genes (Fig. 7*J*). Altogether, these results indicate that the combination of 0602K and Lira improves NASH in this mouse model.

### MSDC-0602K decreases insulinemia and improves pancreas insulin content in MS-NASH mice

**Figure 8:**
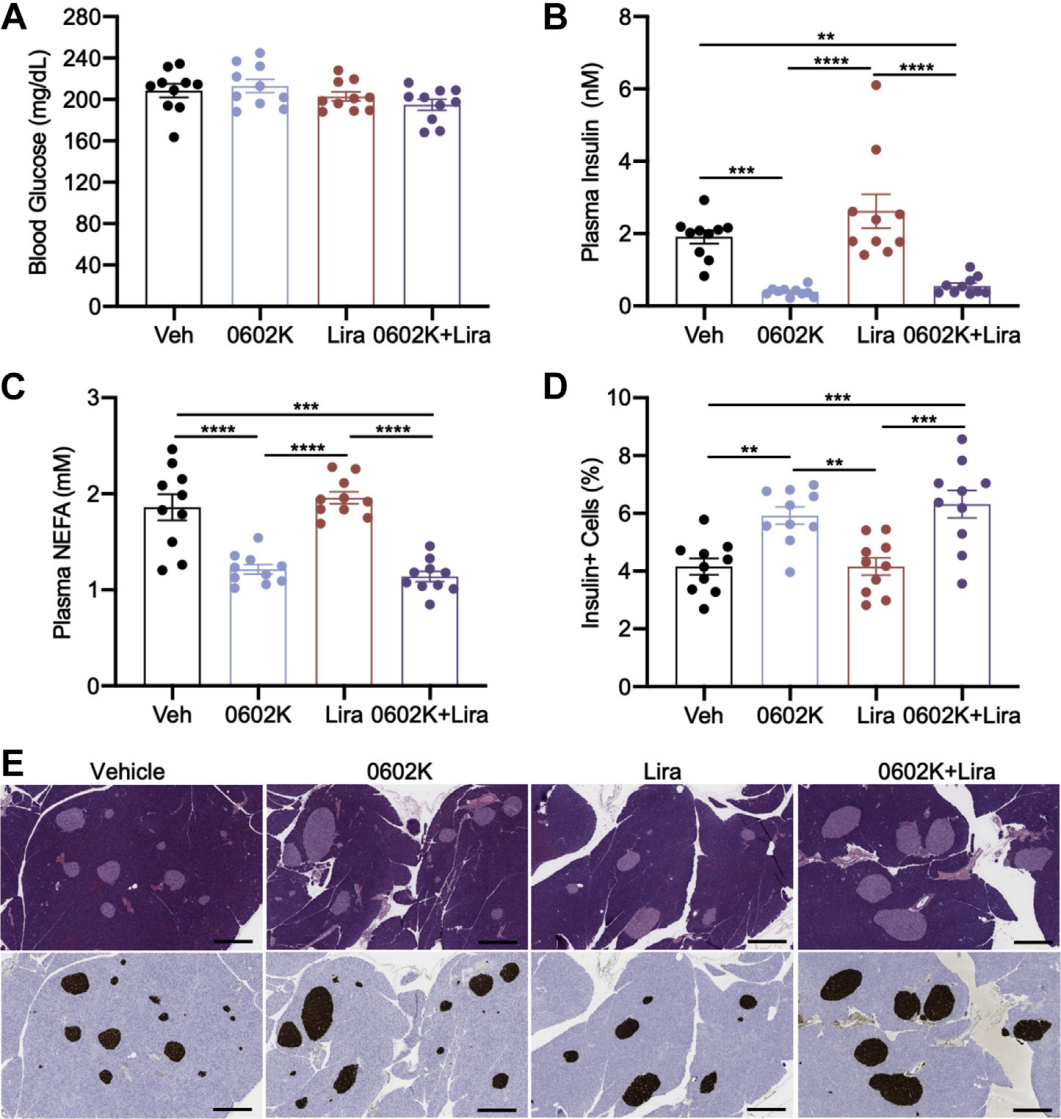
**MSDC-0602K improves insulinemia and islet pancreatic insulin content in MS-NASH mice.***A*–*C*, blood glucose concentrations, plasma insulin concentrations, and plasma nonesterified fatty acids (NEFA). *D* and *E*, percentage of insulin-positive cells in pancreas sections and representative pancreas H&E histology and insulin immunohistochemistry images (scale bars, 200 μm). n = 10 for all groups. All data are mean ± SEM. Individual data points represent a single mouse. Ordinary one-way ANOVA with Tukey’s multiple comparison test: ∗∗*p* < 0.01, ∗∗∗*p* < 0.001, and ∗∗∗∗*p* < 0.0001.

0602K, Lira, or 0602K+Lira treatment did not reduce blood glucose in these MS-NASH mice (Fig. 8*A*). However, 0602K or 0602K+Lira treatment strongly reduced plasma insulin (Fig. 8*B*). Plasma NEFAs were also significantly reduced by 0602K or 0602K+Lira treatment (Fig. 8*C*), suggesting improved insulin action and decreased adipose lipolysis. Finally, insulin immunohistochemistry identified a greater number of insulin+ cells in 0602K- or 0602K+Lira-treated pancreata (Fig. 8, *D* and *E*). Thus, similar to the *db/db* study, these results suggest that the improved insulin sensitivity with 0602K increases islet insulin content.

## Discussion

The overall goal of these present studies was to assess whether combination of a novel TZD insulin sensitizer and a GLP-1RA would improve diabetes and NASH better than either individual therapy. Studies in *db/db* mice suggested that combining MSDC-0602K and liraglutide did indeed provide more significant improvement in glucose tolerance. Glycemia, insulinemia, and insulin sensitivity were greatly improved with 0602K and not further improved by 0602K+Lira treatment. 0602K also improved pancreatic insulin content, and 0602K+Lira further increased islet insulin content. In MS-NASH mice, although plasma biomarkers and some aspects of NASH histology were improved by each treatment, more significant improvements were achieved with combination 0602K+Lira. A secondary goal of these studies was to test if the weight loss associated with liraglutide could prevent the weight gain associated with TZDs. In the *db/db* study, 0602K+Lira-treated mice displayed similar slight weight gain as 0602K-treated mice; however, there was some attenuation of weight gain from combined 0602K+Lira treatment in the MS-NASH study. A study in diabetic rats using a rather low dose of Pioglitazone and double the dose of Lira as used in the current study also observed the greatest improvement in glucose tolerance with Pioglitazone+Lira, and Lira-treated rats lost weight, whereas Pioglitazone+Lira did not attenuate the Pioglitazone-induced weight gain (27). However, a study of Pioglitazone+Lira in *db/db* mice did observe attenuation of the Pioglitazone-induced weight gain with combined Lira treatment (28).

The strongest effects observed in our *db/db* study were the complete correction of glycemia and reduction in insulinemia with 0602K. This decrease in insulinemia with TZDs has been well described in both humans and animal models of insulin resistance and diabetes (29, 30, 31). The presiding dogma is that improved insulin sensitivity indirectly reduces the need for insulin hypersecretion, and thus TZDs can improve beta cell insulin content and function (3, 31, 32, 33). However, it has recently been recognized that traditional and PPARγ-sparing TZDs can bind and inhibit the MPC (8, 9, 10), and pyruvate import into the mitochondria *via* the MPC plays an important role in beta cell glucose sensing and GSIS (26, 34). This raises the possibility that MPC inhibition with TZDs may directly inhibit beta cell insulin secretion, and indeed there are reports of acute TZD treatment decreasing GSIS (35, 36). In this study, however, MSDC-0602K did not reduce GSIS *in vivo* or in isolated islets (Fig. 4*C*), and 0602K was still able to reduce plasma insulin and C-peptide concentrations in mice lacking MPC expression in beta cells (Fig. 4, *E* and *F*). A similar TZD molecule, MSDC-0160, was also found to have no effect on GSIS but improved insulin content and beta cell phenotype in isolated human islets (37). Of interest, MPC inhibition with the tool-compound UK-5099 appears to alleviate the metabolic stress of islets cultured in high glucose (38). Finally, the preserved islet insulin content with TZDs can lead to enhanced insulin secretion during glucose challenge (31, 32), and this was noted with 0602K or 0602K+Lira treatment in this study (Fig. 5*A*). Altogether, these findings suggest that TZDs do not directly inhibit GSIS, but rather they improved insulin sensitivity indirectly by reducing the need for excessive chronic insulin secretion.

Although we observed similar decreases in insulinemia with 0602K or 0602K+Lira in the MS-NASH model of nonalcoholic steatohepatitis, the main goal of this experiment was to analyze fatty liver pathology. All treatments improved plasma ALT and AST, as well as certain aspects of liver histology, with the combination of 0602K+Lira typically resulting in more significant improvements (Fig. 6, *C*–*F* and Fig. 7, *E*–*I*). Similar to our previous study in which we fed C57BL/6J mice a NASH-inducing diet (16), 0602K treatment did not improve hepatic triglycerides and steatosis (Fig. 7, *C*, *E* and *F*). Conversely, steatosis was improved by 0602K in humans with NASH (13). Our previous study identified reductions in histologic fibrosis with 0602K (16), which were not observed in the current model, even with 0602K+Lira combination. Yet both the current and previous studies identified reduced expression of genes related to hepatic stellate cell activation and fibrosis, and these were not further reduced by 0602K+Lira (Fig. 7*J*). Overall, despite no improvements in hepatic steatosis, total NAFLD histologic scores were improved by both therapies, and most significantly by the combination of 0602K+Lira.

There were a limited number of analyses that displayed additive improvement with combined 0602K+Lira treatment. Namely, glucose tolerance in the *db/db* mice and liver weights and combined NAFLD activity scores in the MS-NASH mice were the main endpoints for which 0602K+Lira outperformed 0602K-only treatment. This could be due to a rather low dose of liraglutide used in these studies (0.2 mg/kg every other day), compared with other rodent studies that use similar doses with daily or even twice daily injections (28, 39, 40). However, we are confident that we provided an effective dose of liraglutide as Lira-treated animals displayed weight loss, increased plasma insulin concentrations, and improved glycemia. It is most surprising that, even though Lira increased insulinemia, combination with 0602K resulted in similar suppression of insulinemia compared with 0602K-treated mice but with improved effects from the 0602K+Lira combination on glucose tolerance.

In conclusion, although MSDC-0602K improved many aspects of insulin resistance and NASH by itself, glucose tolerance and several aspects of NASH were more significantly improved by combining 0602K and liraglutide. In the MS-NASH study, Lira attenuated the weight gain associated with 0602K, but this was not the case in *db/db* mice. This study also clarifies that TZDs likely reduce insulinemia not by directly inhibiting GSIS in beta cells but indirectly by improving peripheral insulin sensitivity and decreasing the need for heightened secretion. It remains unclear why MPC inhibition with TZDs does not reduce GSIS unlike genetic MPC deletion (16). Nonetheless, these studies suggest that combining insulin sensitizer and GLP-1RA therapies may better improve diabetes and fatty liver disease and potentially attenuate TZD-induced weight gain.

## Experimental procedures

All experiments and procedures were approved by the Institutional Animal Care and Use Committee of Saint Louis University and conform to NIH guidelines for the care and use of laboratory animals (41). All mice were housed in standard rodent caging at 20 to 25 °C with *ad libitum* access to food and water with a light cycle from 6:00 AM to 6:00 PM.

### *db/db* Mouse studies

Five-week-old male *db/db* mice on C57BL/6J background and age/sex-matched *db/+* control mice were purchased from The Jackson Laboratory (B6.BKS(D)-*Lepr*^db^/J, stock #000697). Mice were provided *ad libitum* access to water and chow (Laboratory Rodent Diet 20 (5L0B), LabDiet). At 9 weeks of age, blood glucose was measured by nick of the tail vein and measuring with a Contour Next glucometer (Ascensia Diabetes Care). The *db/db* mice were then divided into treatment groups based on averaged body weights and blood glucose concentrations: Vehicle (Veh), 30 mg/kg MSDC-0602K (0602K) gavage daily, 0.2 mg/kg liraglutide (Lira) s.c. injection every other day, or combined 0602K+Lira. Lira was obtained from MedChemExpress (HY-P0014). Vehicle gavage solution was 1% low-viscosity carboxymethylcellulose, 0.1% Tween-80, and 1% dimethyl sulfoxide (DMSO). A 0.9% NaCl solution was vehicle for s.c. injection. *db/+* and *db/db* Veh mice received both the gavage and s.c. injection vehicles. 0602K-treated mice also received vehicle s.c. injection, whereas Lira-treated mice also received gavage vehicle. After 3 weeks of treatment, mice were euthanized by CO_2_ asphyxiation and blood was collected by cannulation of the inferior vena cava, placed into an EDTA-containing tube, and centrifuged at 8000*g* for 8 min at 4 °C to collect plasma. Tissues were dissected, blotted dry, frozen in liquid nitrogen, and stored at −80 °C. Pieces of liver and pancreas were placed in neutral buffered formalin for histological evaluation.

### Glucose and insulin tolerance tests

For the GTT, at 8:00 AM mice were weighed and fasted. Four hours later, mice were injected i.p. with 1 g/kg D-glucose in 0.9% NaCl immediately after measuring blood glucose (T = 0) by a nick to the tail vein and a Contour Next glucometer (Ascensia Diabetes Care). Blood glucose was monitored at 15, 30, 60, 90, and 120 min post injection by removing the scab from the tail. In a subset of mice, ∼50 to 60 μl of blood was removed from the tail nick collected by a heparinized capillary tube both before (T = 0) and 30 min following 1 g/kg glucose i.p. injection. Plasma insulin was measured as stated below. For insulin tolerance test, mice were weighed and fasted at 9:00 AM and 4 h later injected i.p. with 0.75 U/kg insulin (Humulin) in 0.9% NaCl, with blood glucose measured from a lateral nick of the tail vein at T = 0, 30, 60, 90, and 120 min post injection.

### Western blotting for insulin signaling

A subset of mice was injected i.p. with 0.9% NaCl or 5 mU/g insulin in 0.9% NaCl and sacrificed 10 min post injection. Tissues were harvested and snap frozen in liquid nitrogen and stored at −80 °C until analyzed. Approximately 50 mg of liver tissue was homogenized using a bead homogenizer (Mini-Beadbeater, Biospec Products Inc) in an NP-40-based lysis buffer (15 mM NaCl, 25 mM Tris base, 1 mM EDTA, 0.2% NP-40, 10% glycerol) supplemented with 1X complete protease inhibitor cocktail and phosphatase inhibitors (1 mM Na_3_VO_4_, 1 mM NaF, and 1 mM PMSF). Protein concentrations were measured by MicroBCA (ThermoFisher Scientific), and 50 μg of protein was electrophoresed on precast Criterion 4% to 15% polyacrylamide gels (Bio-Rad) and transferred onto 0.45 μm Immobilon polyvinyldifluoride membranes (MilliporeSigma). Membranes were then blocked in 5% bovine serum albumin (BSA) in Tris-buffered saline with Tween-20 (TBST) for 1 h. Primary antibodies were then used at 1:1000 in 5% BSA-TBST overnight while rocking at 4 °C. Antibodies for phosphorylated AKT S473 and total AKT were from Cell Signaling (4060 and 4691, respectively), whereas antibody for α-Tubulin was from Sigma (T5168). After primary antibody incubation, membranes were washed with TBST and probed with near-IRDye secondary antibodies (926-32213 and 926-68072) in 5% BSA-TBST for 1 h, washed, and then developed on a LiCor Odyssey imager. AKT activation by insulin was quantified by measuring the densitometry of pAKT-S473 and total AKT using LiCor ImageStudio Lite software and calculating the ratio of phosphorylated AKT/total AKT.

### MS-NASH mouse studies

Studies of the MS-NASH mouse model (Jackson Laboratory MSNASH/PcoJ, stock #030888) were performed at CrownBiosciences Inc. Beginning at 9 weeks of age, male MS-NASH mice were fed *ad libitum* with a “Western diet” (D12079B, Research Diets Inc) containing 40% kcal fat, 17% kcal protein, 43% kcal carbohydrate, and 1.5 g/kg cholesterol and *ad libitum* drinking water containing 5% (w/v) fructose. Body weight was measured weekly, and blood was collected *via* the tail vein for measurement of blood glucose and plasma ALT and AST every 4 weeks as described below. Based on average ALT concentrations, mice were divided into four treatments: Veh, 0602K, Lira, or 0602K+Lira, performed identically to the *db/db* studies described above for a duration of 6 weeks. Mice were euthanized by CO_2_ asphyxiation, and blood was collected *via* cardiac puncture. Tissues were dissected and frozen in liquid nitrogen. Pieces of liver and pancreas were fixed in neutral buffered formalin for histological examination.

### RIPCreMPC2−/− mouse studies

Generation of *Mpc2* floxed mice and RIPCreMPC2−/− pancreatic beta cell MPC2−/− mice on the C57BL/6J background was reported previously (10, 26). For these studies, 6-week-old male RIPCreMPC2−/− and littermate fl/fl control mice were fed 60% high-fat diet for 10 weeks (D12492, Research Diets Inc). Based on body weight, mice were divided into two single gavage treatments: Vehicle or 30 mg/kg 0602K. Sixteen hours post gavage, mice were euthanized by CO_2_ asphyxiation. Blood was collected from the inferior vena cava into an EDTA-coated tube and processed as above, and plasma was stored at −80 °C.

### Seahorse analysis

Beta TC6 insulinoma cells were purchased from ATCC (ATCC-CRL-11506) and were cultured in Dulbecco's modified Eagle's medium (DMEM) +15% FBS and 1% Pen/Strep in a 5% CO_2_ incubator. The day prior to assay, cells were trypsinized and plated onto a Seahorse 96-well XFe plate at a density of 15,000 cells per well. On the next day, 1 h prior to assay, cells were changed to Seahorse DMEM medium with either DMSO vehicle, 20 μM MSDC-0602K, or 5 μM UK-5099 and placed in a CO_2_-free incubator. The assay DMEM medium contained 20 mM glucose and 2 mM pyruvate. After the 1-h preincubation, cells were assayed with a “mitochondrial stress test” in a Seahorse XFe96 bioanalyzer (Agilent). Injected compounds were 2 μM oligomycin, 2 μM FCCP, and 1 μM rotenone and antimycin A. At baseline and after each injection both oxygen consumption rates and extracellular acidification rates (glycolysis) were measured three times with 2-min mixing periods between each measurement.

### Pancreatic islet isolation and glucose-stimulated insulin secretion assay

Wildtype male and female C57BL/6J mice were obtained (Jackson Laboratory, stock #000664) and maintained on normal chow. Between 10 and 20 weeks of age, mice were euthanized by 3% to 5% isoflurane inhalation and cervical dislocation. Islets were isolated similar to previously described procedures (26, 42), after perfusion of the pancreas through the common bile duct with 5 to 10 ml of calcium-free Hanks-buffered saline solution supplemented with 0.4 mg/ml Type V collagenase (C9263, Sigma). After overnight culture, islets were treated with either 1 or 23 mM glucose and either DMSO vehicle or 10 μM 0602K in a 37 °C incubator for 1 h. Insulin concentration of the supernatants was measured with a mouse/rat insulin ELISA (EZRMI-13K, EMD Millipore).

### Plasma hormone, metabolite, and ALT and AST assays

Plasma fructosamine levels were measured with a colorimetric kit (K450-100, BioVision Inc). Plasma insulin and C-peptide were measured with mouse/rat ELISAs (EZRMI-13K and EZRMCP2-21K, respectively, EMD Millipore). Plasma NEFAs were measured by enzymatic colorimetric assay (NEFA-HR(2), FUJIFILM Wako). Plasma triacylglycerol (TAG) and cholesterol were measured with colorimetric assays (TR22421 and TR13421, respectively, ThermoFisher Scientific). Plasma ALT and AST concentrations were measured with kinetic spectrophotometric assays (A524-150 and A559-150, respectively, Teco Diagnostics).

### Liver TAG and glycogen assays

Liver TAG concentrations were measured as described (16). Frozen tissue, 40 to 140 mg, was homogenized using a bead homogenizer (Mini-Beadbeater, Biospec Products Inc) in 0.9% NaCl at a volume to provide 0.1 mg liver/μl. Liver homogenate was combined (1:1) with 1% sodium deoxycholate, vortexed, and placed at 37 °C for 5 min to solubilize lipids. TAG was then measured by colorimetric assay (TR22421, ThermoFisher Scientific).

Liver glycogen was measured using previously described methods (43). Liver tissue, 20 to 75 mg, was boiled in 300 μl of 30% KOH at 100 °C for 30 min. Tubes were cooled on ice, and 100 μl of 2% Na_2_SO_4_ and 800 μl of 100% EtOH was added and the tubes were vortexed. Tubes were boiled for 5 min and centrifuged at 16,000*g* for 5 min; then the supernatant was aspirated. The pellet was dissolved in 1 ml of 80% EtOH and recentrifuged at 16,000*g* for 5 min. The final pellets were resuspended in 200 μl of 0.3 mg/ml amyloglucosidase (Sigma) in 0.2 M sodium acetate. Serial dilutions of 10 mg/ml oyster glycogen (Sigma) were prepared as standards. Samples and standards were incubated in a 40 °C water bath for 3 h and then diluted 1:1 with H_2_O; 5 μl of each was added to a 96-well plate. Two hundred microliters of glucose assay buffer (0.3 M triethanolamine, pH∼7.5, 4 mM MgCl_2_, 2 mM ATP, 0.9 mM NADP+, and 5 μg/ml glucose-6-phosphate dehydrogenase) was added to each well, and absorbance was measured at 340 nm. Hexokinase (1 μg; Sigma) was then added to each well, the plate incubated at room temperature in the dark for 30 min, and absorbance remeasured at 340 nm.

### Pancreas and liver histology and immunohistochemistry analyses

Formalin-fixed liver and pancreas sections were embedded in paraffin blocks, processed routinely, and sectioned onto glass slides. Liver slides were stained for H&E and picrosirius red and were evaluated by a histopathologist blinded to mouse treatment and group designations. Liver slides were scored for NAFLD activity (combined scores of steatosis, inflammation, and hepatocyte ballooning) and fibrosis similar to human biopsy scoring (44).

For the *db/db* study, unstained pancreas slides were used for immunofluorescence as described (26). Slides were rehydrated then permeabilized with 1 mg/ml trypsin (T7168, Sigma) in H_2_O for 25 min at room temperature. Slides were washed 2 × 5 min with 0.2% NP40-PBS and blocked with 0.2% NP40-PBS containing 3% BSA for 30 min at room temperature. Slides were incubated with primary antibodies overnight at 4 °C in a humidity chamber. Primary antibodies were guinea pig anti-insulin polyclonal and mouse anti-glucagon monoclonal, both 1:100 (ab7842 and ab10988, respectively, Abcam). On the following day, slides were washed 3 × 5 min with 0.2% NP40-PBS, then incubated with secondary antibodies for 2 h at room temperature. Secondary antibodies were goat anti-guinea pig, Alexa Fluor 488 and goat anti-mouse, Alexa Fluor 594 (A11073 and A21125, respectively, ThermoFisher Scientific). Slides were washed 3 × 5 min with 0.2% NP40-PBS, and glass coverslips were mounted with ProLong Diamond with DAPI (P36971, ThermoFisher Scientific). Slides were imaged on a Leica DM5500B fluorescence microscope with Leica DFC365 FX camera (Leica Microsystems Inc). Intensity of the green insulin fluorescence of pancreatic islets relative to nearby exocrine pancreas was measured using NIH ImageJ software.

For the MS-NASH study, pancreas slides were stained with H&E or prepared for insulin immunohistochemistry by performing heat-induced epitope retrieval using EnVision FLEX Target Retrieval Solution, low pH∼6 (Dako, K8005; Agilent), with preheating to 80 °C and increased temperature to 95 °C for 20 min after slides were added. Slides were then incubated in 3% H_2_O_2_ for 5 min and incubated with rabbit anti-Insulin antibody, 1:100, for 45 min (#4590, Cell Signaling Technology). Slides were then washed, and secondary antibody EnVision+ anti-rabbit labeled polymer-HRP was applied for 30 min (Dako, K4003; Agilent), followed by DAB+ chromogen solution for 5 min. Slides were then rinsed in water and counterstained with modified Harris hematoxylin (Dako, S3301; Agilent). Slides were covered with coverslip and scanned at 20X on an Aperio Scanscope XT (Leica Biosystems), and insulin+ cells were quantified by HALO software (Indica Labs).

### RNA isolation, cDNA synthesis, and quantitative RT-PCR analyses

Frozen liver tissue, 5 to 20 mg, was homogenized in 1 ml RNA-STAT reagent (Tel-Test) with isopropanol and EtOH precipitation. RNA pellets were resuspended in 200 μl water and assessed by Nanodrop (ThermoFisher Scientific). RNA, 1 μg, was reverse transcribed into cDNA by Superscript VILO kit (ThermoFisher Scientific), using an Applied Biosystems 2720 Thermal Cycler (ThermoFisher Scientific). qPCR was performed for all samples in duplicate with Power SYBR Green using an Applied Biosystems QuantStudio-3 real-time thermocycler (ThermoFisher Scientific). Target gene Ct values were normalized to reference gene (*Rplp0*) Ct values by the 2^−ΔΔCt^ method. Oligonucleotide primer sequences are listed in electronic supplementary material Table S1.

### Statistics

All data are expressed as means ± SEM, with individual data points shown as dot plots, or curves over time displayed as means ± SEM. Individual data points represent a single mouse. No data were excluded from any study measurements. Investigators were not blinded to genotype or treatment. The *db/db* study was performed with three separate cohorts/replicates of mice and combined. For liver histology analysis of the MS-NASH studies, the pathologist was blinded to mouse number and treatment groups. The MS-NASH study was performed with a single cohort of mice. Statistical analyses were performed using GraphPad Prism 8 (GraphPad) using ordinary one-way ANOVA and Tukey’s multiple comparisons tests, and *p* < 0.05 was considered statistically significant.

## Data availability

All data generated during these studies are included in the text, figures, and tables of this article and electronic supplementary material. Source data or materials will be supplied by the corresponding author with reasonable request.

## Supporting information

This article contains supporting information.

## Conflict of interest

J. R. C. is an employee, chief scientific officer, and stockholder of Cirius Therapeutics, which is developing MSDC-0602K for NASH. All other authors declare no relationships or conflicts of interest.
