# Supplementary material for: Novel insulin sensitizer MSDC-0602K improves insulinemia and fatty liver disease in mice, alone and in combination with liraglutide
Source: J Biol Chem. 2021 May 20;296:100807. doi: 10.1016/j.jbc.2021.100807 (PMC8192871; doi:10.1016/j.jbc.2021.100807)
Supplement: Supplemental Table S1 [file mmc1.pdf]

Supplementary Table 1. Primer oligonucleotide sequences used for qRT-PCR of mouse liver.

Gene	Forward 5'-3'	Reverse 5'-3'	Amplicon Length
Acta2	gtc cca gac atc agg gag taa	tcg gat act tca gcg tca gga	102
Col1a1	gct cct ctt agg ggc cac t	cca cgt ctc acc att ggg g	103
Col1a3	ctg taa cat gga aac tgg gga aa	cca tag ctg aac tga aaa cca cc	144
Rplp0	gca gac aac gtg ggc tcc aag cag at	ggt cct cct tgg tga aca cga agc cc	190
Spp1	atc tca cca ttc gga tga gtc t	tgt agg gac gat tgg agt gaa a	79
Tgfb1	ctc ccg tgg ctt cta gtg c	gcc tta gtt tgg aca gga tct g	133
Timp1	cca gag ccg tca ctt tgc tt	agg aaa agt aga cag tgt tca ggc tt	126
